# Supplementary material for: Obstructive Tracheobronchitis in Influenza-Associated Pulmonary Aspergillosis
Source: Diagnostics (Basel). 2024 Jul 28;14(15):1628. doi: 10.3390/diagnostics14151628 (PMC11311288; doi:10.3390/diagnostics14151628)

Figure S2. A repeated CT scan on the day following intubation and ICU admission (23/1/2024) revealed bronchitis, bilateral patchy opacities, and small nodular lesions in the lung parenchyma. There were significant artifacts, and the findings indicated substantial progression compared to the scan performed four days earlier (19/1/2024).

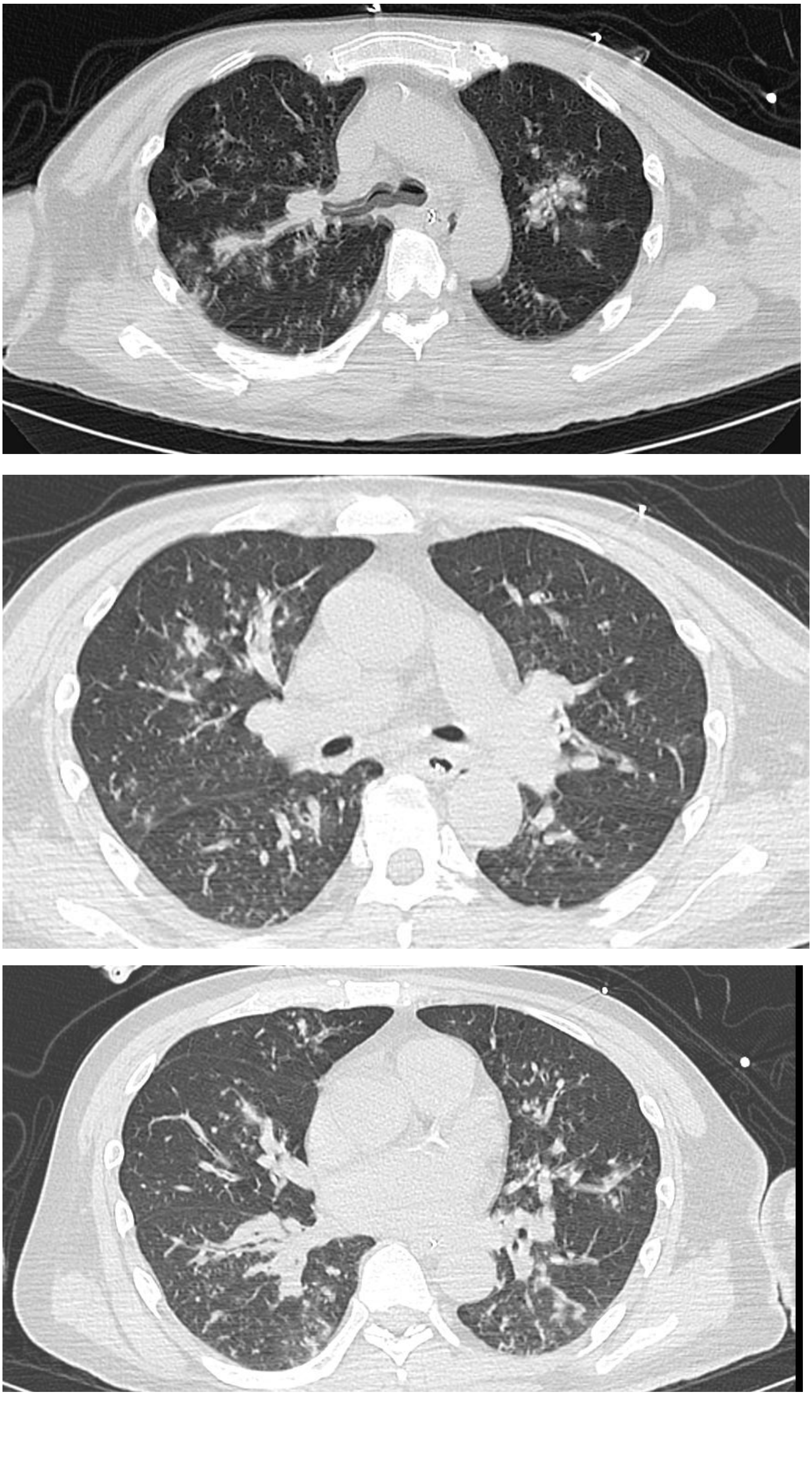

Supplement: Supplementary file 1 [file diagnostics-14-01628-s001.zip › supplement file -Figure S2.pdf]
