# Supplementary material for: Obstructive Tracheobronchitis in Influenza-Associated Pulmonary Aspergillosis
Source: Diagnostics (Basel). 2024 Jul 28;14(15):1628. doi: 10.3390/diagnostics14151628 (PMC11311288; doi:10.3390/diagnostics14151628)
Supplement: Supplementary file 1 [file diagnostics-14-01628-s001.zip › supplement file -Figure S3.pdf]

Figure S3. After two weeks of antifungal treatment, repeated bronchoscopy revealed significant improvement of airway obstruction, with scaring of the mucous, dilation of airways and remaining pseudomembrane and ascars (Left). Compared to bronchoscopy upon ICU admission (Right).

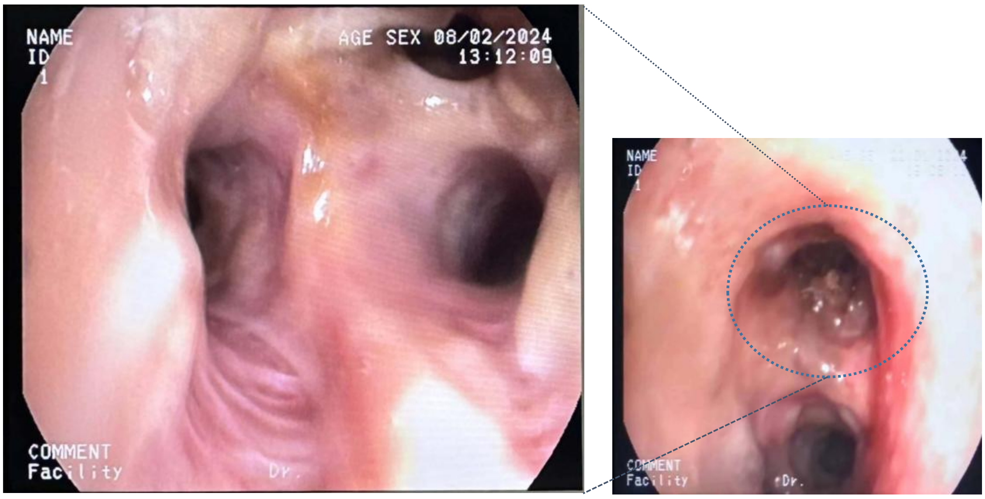

A. Opening of the right upper lobe

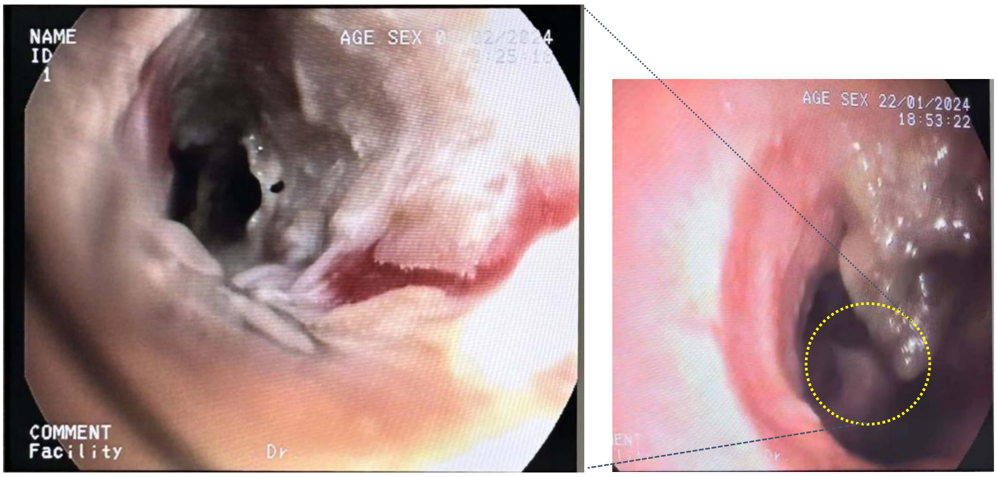

B. Left main bronchus

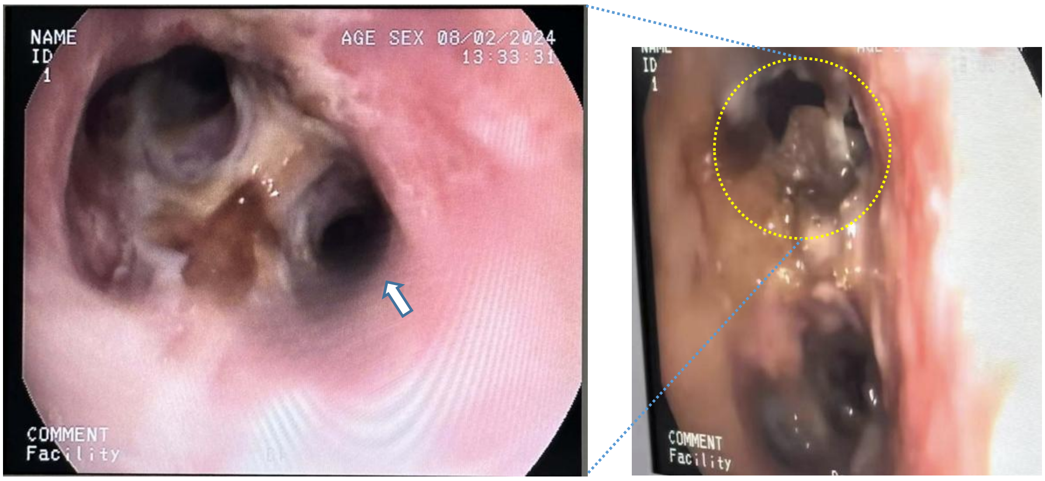

C. Opening of the left upper lobe; White arrow: Lingular segment of the left upper lobe

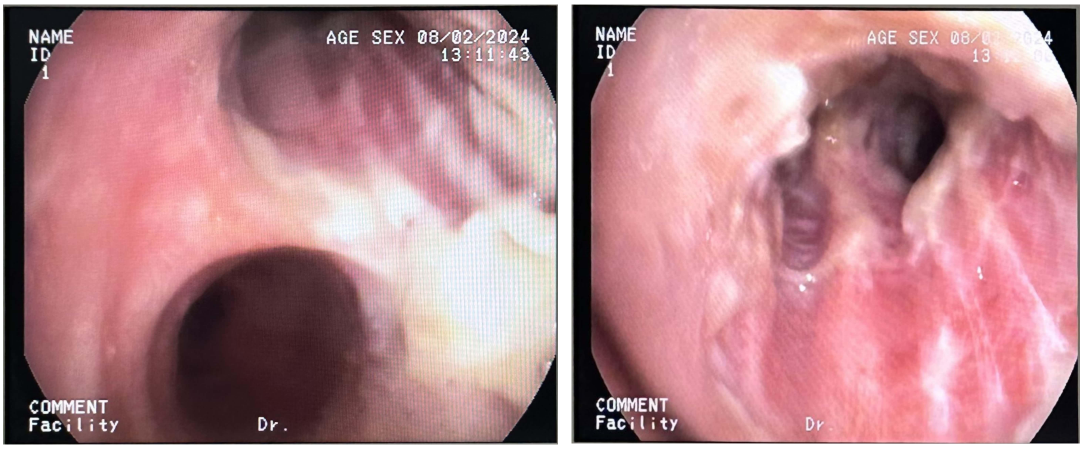

D. Carina (left) and opening of the right middle lobe (right)
