# Supplementary material for: Obstructive Tracheobronchitis in Influenza-Associated Pulmonary Aspergillosis
Source: Diagnostics (Basel). 2024 Jul 28;14(15):1628. doi: 10.3390/diagnostics14151628 (PMC11311288; doi:10.3390/diagnostics14151628)

Figure S1. A CT scan done in ED three days prior the bronchoscopy revealed only bronchitis, with no parenchymal lung changes.

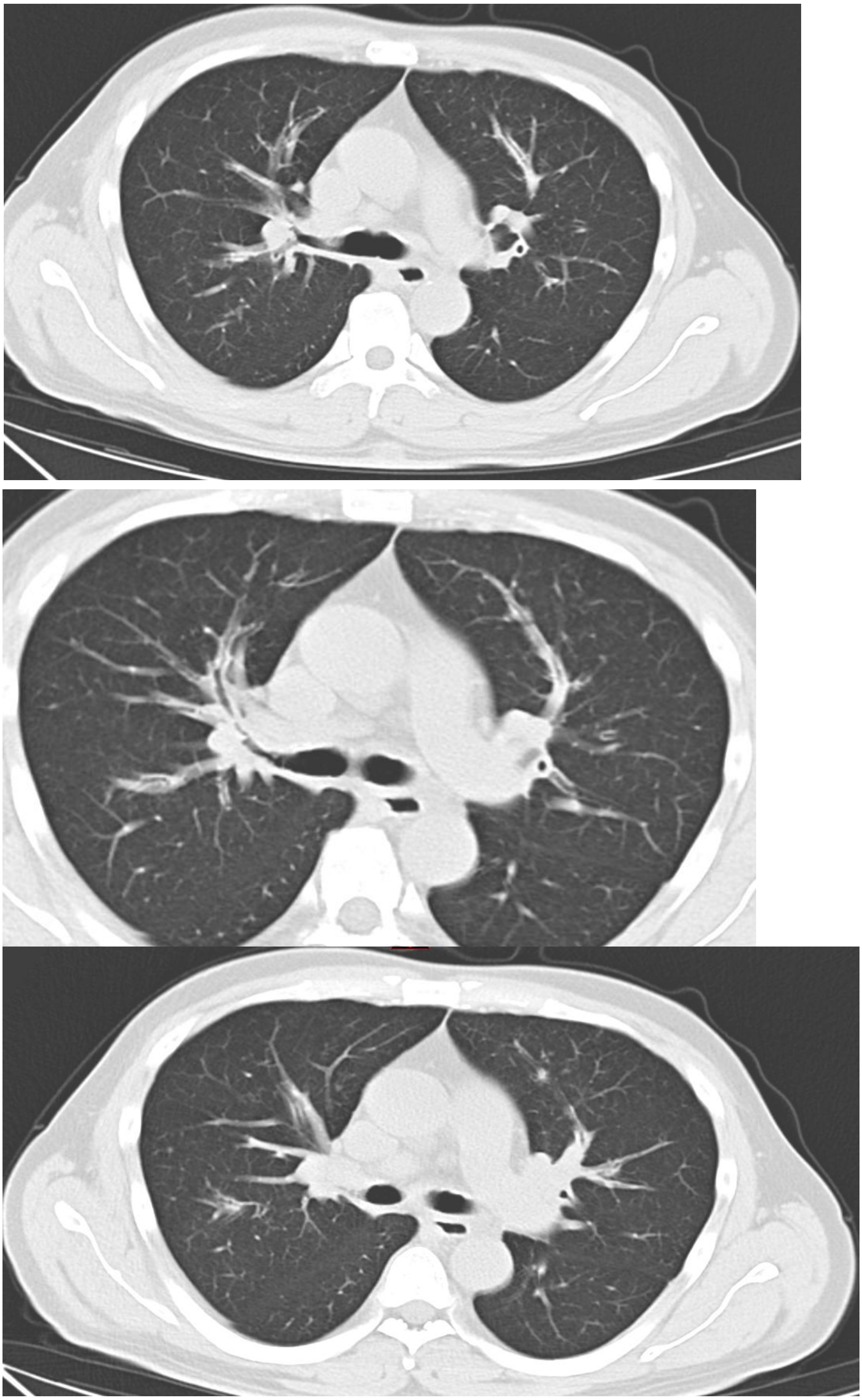

Supplement: Supplementary file 1 [file diagnostics-14-01628-s001.zip › supplement file- Figure S1.pdf]
